# Viral Ubiquitin Ligase Stimulates Selective Host MicroRNA Expression by Targeting ZEB Transcriptional Repressors

**DOI:** 10.3390/v9080210

**Published:** 2017-08-07

**Authors:** Gabriel Lutz, Igor Jurak, Eui Tae Kim, Ju Youn Kim, Michael Hackenberg, Andrew Leader, Michelle L. Stoller, Donna M. Fekete, Matthew D. Weitzman, Donald M. Coen, Angus C. Wilson

**Affiliations:** 1 Department of Microbiology, New York University School of Medicine, New York, NY 10016, USA; Gabriel.Lutz@med.nyu.edu (G.L.); jyk294@gmail.com (J.Y.K.); 2 Department of Biological Chemistry and Molecular Pharmacology, Harvard Medical School, Boston, MA 02115, USA; igor.jurak@biotech.uniri.hr (I.J.); Andrew.Leader@icahn.mssm.edu (A.L.); don_coen@hms.harvard.edu (D.M.C.); 3 Department of Biotechnology, University of Rijeka, 51000 Rijeka, Croatia; 4 Department of Pathology and Laboratory Medicine, University of Pennsylvania Perelman School of Medicine and The Children’s Hospital of Philadelphia, Philadelphia, PA 19104, USA; kime2@email.chop.edu (E.T.K.); weitzmanm@email.chop.edu (M.D.W.); 5 Department of Genetics, Computational Genomics and Bioinformatics Group, University of Granada, Granada 18071, Spain; hackenberg@go.ugr.es; 6 Department of Biological Sciences, Purdue University, West Lafayette, IN 47907, USA; mstoller2@gmail.com (M.L.S.); dfekete@purdue.edu (D.M.F.)

**Keywords:** herpes simplex virus, HSV-1, microRNA, miR-183, miR-96, miR-182, ICP0, E3 ubiquitin ligase, ZEB, host shutoff

## Abstract

Infection with herpes simplex virus-1 (HSV-1) brings numerous changes in cellular gene expression. Levels of most host mRNAs are reduced, limiting synthesis of host proteins, especially those involved in antiviral defenses. The impact of HSV-1 on host microRNAs (miRNAs), an extensive network of short non-coding RNAs that regulate mRNA stability/translation, remains largely unexplored. Here we show that transcription of the miR-183 cluster (miR-183, miR-96, and miR-182) is selectively induced by HSV-1 during productive infection of primary fibroblasts and neurons. ICP0, a viral E3 ubiquitin ligase expressed as an immediate-early protein, is both necessary and sufficient for this induction. Nuclear exclusion of ICP0 or removal of the RING (really interesting new gene) finger domain that is required for E3 ligase activity prevents induction. ICP0 promotes the degradation of numerous host proteins and for the most part, the downstream consequences are unknown. Induction of the miR-183 cluster can be mimicked by depletion of host transcriptional repressors zinc finger E-box binding homeobox 1 (ZEB1)/δ-crystallin enhancer binding factor 1 (δEF1) and zinc finger E-box binding homeobox 2 (ZEB2)/Smad-interacting protein 1 (SIP1), which we establish as new substrates for ICP0-mediated degradation. Thus, HSV-1 selectively stimulates expression of the miR-183 cluster by ICP0-mediated degradation of ZEB transcriptional repressors.

## 1. Introduction

MicroRNAs (miRNAs) function as post-transcriptional regulators of gene expression, limiting protein expression through a combination of mRNA destabilization and translational inhibition [1]. The majority of cellular mRNA transcripts are targeted by at least one, and more often by many, miRNAs creating an integrated network that helps coordinate the output of genes within biological pathways [2]. MiRNAs also contribute to viral pathogenesis, either participating in antiviral defenses or by enhancing viral replication and stabilizing competing programs of viral gene expression [3,4,5,6].

Several virus families produce their own viral miRNAs, with the herpesviruses providing the most prominent examples [7]. In the case of the prototype α-herpesvirus, herpes simplex virus 1 (HSV-1, human alphaherpesvirus 1), several viral miRNAs are preferentially expressed in latency, a persistent but largely silent infection restricted to neurons, while others are preferentially expressed during productive (lytic) infection [8,9,10,11]. Interestingly, one HSV-1 miRNA, miR-H2, and host miRNA, miR-138, both target a key viral regulatory factor, ICP0, with the host miRNA being a more effective suppressor in both cultured cells and ganglia of infected mice [12,13,14,15]. Additional examples of cooperation between viral and host miRNAs to benefit viral replication or latency have been described for other herpesviruses, including human cytomegalovirus (HCMV) and Epstein–Barr virus (EBV) [16,17,18].

In broad strokes, two general strategies have been uncovered by which viruses alter host miRNA–mRNA networks. The first strategy is to express viral miRNAs that mimic existing cellular miRNAs, allowing the virus to exploit an evolutionarily inflexible network of binding sites in target mRNAs. For example, Kaposi’s sarcoma-associated herpesvirus (KSHV), avian Marek’s disease virus-1 and 2 (MDV-1 and MDV-2), EBV, rhesus lymphocryptovirus, and bovine leukemia virus, a retrovirus, encode mimics of miRNAs, miR-155, or miR-29 that promote B-cell tumorigenesis [19,20,21]. The second strategy is to alter the expression of specific cellular miRNAs. During EBV infection for example, miR-155, is strongly upregulated, allowing the virus to use the pro-oncogenic properties of the host miRNA [17]. Alternatively, some viruses employ various mechanisms to downregulate cellular miRNAs. Herpesvirus saimiri expresses high levels of the H. saimiri U-rich RNAs (HSURs) 1 and 2 non-coding RNAS that hybridize through sequence complementarity to specific target miRNAs and promote their rapid degradation [22]. Additionally, HCMV infection reduces the levels of miR-100, and miR-101, which target components of the mammalian target of rapamycin (mTOR) complex, and delivery of unregulated mimics to replace these miRNAs leads to reduced HCMV replication [23].

Suppression of host gene expression, otherwise known as host shutoff, is especially profound in HSV-1 infections and shapes the viral replication program by sharpening the transitions between different kinetic classes of temporally transcribed mRNAs [24,25]. Mechanisms include deregulation of host gene transcription by RNA polymerase II [26,27], reduced export and processing of host mRNAs [28], accelerated mRNA turnover through the action of viral and host ribonucleases [29,30], and suppression of protein synthesis through alterations to the function and abundance of host factors controlling initiation, elongation, and termination of translation [31]. Because miRNAs are widely used to coordinate the output of gene networks by reducing the stability and translation of multiple mRNAs, they would seem to be logical targets for manipulation by viruses [32], but whether miRNAs are induced or shutoff by HSV-1 infection has remained largely unexplored. Here we show that the levels of three cellular miRNAs, miR-96, miR-182, and miR-183, but not most others, are substantially increased during HSV-1 infection of primary cells. As induction of host mRNAs (and miRNAs) by HSV-1 is infrequent, we investigated further and found that this increase is mediated by the viral immediate-early protein ICP0 through its E3 ubiquitin ligase function and reflects transcriptional activation of a single chromosomal locus encoding all three miRNAs. ICP0 regulates a variety of cellular processes, in part by targeting cellular regulatory factors for degradation by the proteasome. As shown here, these targets include ZEB1 and ZEB2, host transcription factors that suppress the transcription of genes involved in maintaining differentiated cell states, including the miR-183/96/182 locus [33,34].

## 2. Materials and Methods

### 2.1. Viruses

The following HSV-1 strains were used: wild type HSV-1 GFP-Us11 (strain Patton) [35] and wild type HSV-1 (strain KOS), 7134 (∆ICP0) and 7134R (ICP0-rescue) viruses originally from Dr. Pricilla Schaffer (Harvard Medical School, Boston, MA, USA) [36], HSV-1 *n*12 (∆ICP4) virus from Dr. Neal DeLuca (University of Pittsburgh School of Medicine, Pittsburgh, PA, USA) [37], HSV-1 *d*27-1 (∆ICP27) virus from Dr. David Knipe (Harvard Medical School, Boston, MA, USA) [38], and HSV-1 HP66 (∆DNA polymerase) [39]. Recombinant adenoviruses (gifts from Drs. William Halford and Pricilla Schaffer [40]) were as follows: Ad.T-n212 (Ad-ΔICP0), Ad.T-ICP4, Ad.T-ICP0, Ad.T-VP16, and Ad.C-rtTA. For adenoviral delivery of the miR-183 cluster, a bifunctional cassette containing the miR-183 family and green fluorescent protein (GFP) [41], was shuffled into the adenoviral vector AD5-CMV-V5-DEST (Invitrogen, Carlsbad, CA, USA) using Gateway^®^ LR Clonase (Invitrogen).

Stocks of the wild type KOS and GFP-Us11 viruses were amplified in Vero cells (American Type Culture Collection (ATCC), Manassas, VA, USA) infected for 1 h at 37 °C at a multiplicity of infection (MOI) of 0.01 in Dulbecco’s Modified Eagle’s medium (DMEM, Invitrogen) supplemented with 2% fetal bovine serum (FBS, Gibco Laboratories, Gaithersburg, MD, USA) and 1% penicillin/streptomycin/l-glutamine (Invitrogen) (*v*/*v*). Infected cultures were incubated at 37 °C in fresh media until cytopathic effects were observed in all cells and then frozen at −80 °C. Virus was liberated by three freeze/thaw cycles with or without sonication and titers determined by plaque assay on Vero cell monolayers. All adenoviruses were propagated in 293A cells cultured in 15 cm dishes and the titer determined by plaque assay in 293A cells (ATCC).

### 2.2. Cell Lines and Infections

293A and 293T cells (both from ATCC) were routinely cultured in DMEM containing 10% FBS and 1% penicillin/streptomycin/l-glutamine (Invitrogen) (*v*/*v*), while Vero cells were cultured in similar media but containing 5% bovine calf serum (BCS). Rat embryonic fibroblast cultures (REFs) were prepared from E18 Sprague Dawley^®^ rat pups (Charles River Laboratories, Kingston, NY, USA) after decapitation and removal of the internal organs. Bodies were dissociated by addition of trypsin for 1hr at 37 °C and the resulting cell suspension was filtered through a 70 μm nylon cell strainer into conical tubes, to which fresh DMEM supplemented with 10% FBS, 1% penicillin/streptomycin/l-glutamine (*v*/*v*) was added to a final volume of 10 mL per pup body and plated onto 10 cm culture dishes. The cells were incubated at 37 °C for 1 h to allow fibroblasts to adhere, supplied with fresh media, and incubated at 37 °C overnight. Cultures were split the following day. Primary normal human diploid fibroblasts (NHDFs, Clonetics™, San Diego, CA, USA) were cultured in DMEM supplemented with 5% FBS, 1% penicillin/streptomycin/l-glutamine (*v*/*v*).

REFs or NHDFs were seeded at a density of 1 × 10^5^ cells/well of a 12-well plate and incubated at 37 °C overnight. The following day, infections were performed using the indicated wild type and mutant HSV-1 viruses at an MOI of 3, for 1 h at 37 °C with rocking every 15 min, after which viral media was replaced with fresh culture media and incubated at 37 °C for 6 h post-infection until RNA and/or protein collection. Infections with Ad.T-ICP0, Ad.T-ICP4, and Ad.T-VP16 were performed at an MOI of 320. All adenoviral infections were performed as co-infections with Ad.C-rtTA, which constitutively expresses the doxycycline-regulated transactivator, at an MOI of 80, for 1 h at 37 °C with rocking every 15 min. After infections, viral media was replaced and adenoviral samples were supplemented with fresh culture media containing doxycycline (2 μg/mL). Adenoviral samples were incubated at 37 °C until RNA and/or protein collection 48 h post-infection.

Coinfection of Vero cells with HSV-1 ΔICP0 and various adenoviruses was performed as described by Halford et al. [40]. HSV-1 ΔICP0, Ad.T-ICP0, Ad.T-VP16, Ad.C-rtTA, and Ad-183F were each used at an MOI of 10. To allow expression, Ad.T-ICP0 and Ad.T-VP16 were coinfected with Ad.C-rtTA. Infections proceeded for 1 h at 37 °C with rocking every 15 min, after which the media was replaced with fresh media containing 1% carboxymethylcellulose to restrict viral spread. For infections with Ad.T-ICP0 and Ad.T-VP16 infections, doxycycline (2 μg/mL) was also included in the media. Infected cultures were incubated at 37 °C for two days before being fixed for 30 min and stained with crystal violet to visualize plaques formed by HSV-1 ΔICP0 replication.

### 2.3. Preparation and HSV-1 Infection of Superior Cervical Ganglia Neuron Cultures

Primary neuron cultures were isolated from the superior cervical ganglia (SCG) of prenatal rats and cultured in vitro as previously described [42,43,44]. On day 6 in vitro, the culture media was replaced with media containing 100 μM acyclovir (ACV) and the next day infected with HSV-1 (Patton) GFP-Us11 (MOI of 1). After 2 h at 37 °C, the media was replaced with neural basal medium supplemented with 50 ng/mL nerve growth factor (NGF) and 100 µM ACV and maintained for a further seven days, during which time latency is established. Prior to harvest, cultures were monitored for GFP fluorescence indicative of low-level spontaneous reactivation and discarded if positive.

### 2.4. Ethics Statement

This study was carried out in strict accordance with the recommendations laid out by the National Institutes of Health (NIH) Guide for the Care and Use of Laboratory Animals. The protocol for the isolation of rat primary neurons was approved by the Institutional Animal Care and Use Committee (IACUC # 101009) of the New York University Langone Medical Center (Public Health Service (PHS) Assurance of Compliance Number: A3435-01). Rats were euthanized by CO_2_ inhalation prior to ganglia harvest.

### 2.5. RNA Extraction and Reverse Transcription Quantitative Polymerase Chain Reaction

Total RNA was collected from mock and HSV-1-infected cells 6 h post-infection unless stated otherwise, or at 48 h post -infection from adenovirus-infected cells by lysis in 350 μL Buffer RLT per well of a 12-well plate followed by homogenization using QIAshredder spin columns (Qiagen, Waltham, MA, USA). RNA was purified using RNeasy Mini kits (Qiagen) using 100% nuclease-free ethanol for RNA precipitation and omitting steps requiring Buffer RW1. Purified total RNA (500 ng as measured by Nanodrop spectrophotometry (NanoDrop Technologies, Wilmington, DE, USA) was treated with DNase I (New England Biolabs, Ipswich, MA, USA) for 30 min at 37 °C and inactivated by addition of 2 mM ethylenediaminetetraacetic acid (EDTA) and incubation at 75 °C for 10 min. RNA samples were then reverse-transcribed using miScript II RT (Qiagen) and mature miRNA levels were quantified using miScript miRNA assay primers (Qiagen), miScript SYBR Green PCR reagents (Qiagen) and a MyiQ single-color thermal cycler (BioRad, Hercules, CA, USA). Relative levels of mature miRNA were normalized to Let-7a using the ΔΔC_T_ method, with data being represented as the mean of triplicates and their standard error (SEM). The relative abundance of host miRNAs in human foreskin fibroblasts (HFFs) was also measured using a Taqman looped primer RT-PCR assay (Applied Biosystems, Foster City, CA, USA). Prism 6 software (GraphPad, La Jolla, CA, USA) was used for statistical analysis and data presentation.

### 2.6. High-Throughput Small RNA-Sequencing

Small-RNA libraries (TrueSeq small RNA sample preparation kit, Illumina, San Diego, CA, USA) were generated using total RNA, and sequenced using a HiSeq2000 sequencer (Illumina) at the Biopolymers Facility, Departments of Genetics, Harvard Medical School, Boston, MA, USA. Data sets were analyzed using sRNAbench software [45] and aligned against known miRNAs from miRBase release 20 using Bowtie seed alignment, allowing for no mismatches within the first 18 nucleotides.

### 2.7. Immunoblotting

Cells were lysed in sodium dodecyl sulfate (SDS) sample buffer, heat denatured for 5 min, fractionated by 7.5% sodium dodecyl sulfate polyacrylamide gel electrophoresis (SDS-PAGE), and transferred to nitrocellulose membranes (Whatman, Maidstone, UK). Membranes were blocked in a 5% milk solution in Tris Buffered Saline with Tween^®^ 20 (TBS-T) for 1 h at room temperature and incubated overnight at 4 °C with various primary antibodies: α-ICP0 (1:1000, Abcam, Cambridge, MA, USA), α-ICP4 (1:1000, Abcam), α-ICP8 (1:1000, Abcam), α-ICP27 (1:1000, Abcam), α-VP16 (1:1000, Sigma-Aldrich, St. Louis, MO, USA), α-ZEB1 (1:500, Novus Biologicals, Littleton, CO, USA), α-SIP1/ZEB2 (1:500, Abcam), β-actin (1:20,000, Sigma-Aldrich), or α-tubulin (1:5000, Sigma-Aldrich). Bound primary antibodies were detected using horseradish peroxidase-conjugated α-mouse or α-rabbit secondary antibodies (both diluted 1:5000, Sigma-Aldrich) with incubation at room temperature for 1 h and visualization by chemiluminescent detection using SuperSignal West Pico ECL substrate (ThermoFisher Scientific, Waltham, MA, USA).

### 2.8. Pharmacological Applications

Proteasome inhibitor MG132 (Sigma) was added to the culture media at a concentration of 40 μM. Cycloheximide (Sigma) was added at 50 μg/mL for the 1 h infection period and the subsequent post-infection incubation time until RNA collection. Doxycycline (Sigma) was added at a final concentration of 2 μM after adenoviral infections.

### 2.9. Immunofluorescence Microscopy

Cells were seeded onto coverslips at a density of 1 × 10^5^ cells per well of a 12 well culture plate, and infected the next day with virus as described above. For imaging, cells were fixed in 4% paraformaldehyde for 20 min, permeabilized with 0.1% Triton X-100 diluted in PBS for 30 min, and blocked in 3% bovine serum albumin (BSA) in TBS-T for 30 min, before being incubated with α-ICP0 antibody (1:200, Abcam) for 1 h at room temperature, followed by several TBS-T washes. Bound primary antibody was detected using an AlexaFluor-568 secondary antibody (1:100, Invitrogen) with incubation for 1 h and counter stained with DAPI (1:100, ThermoFisher Scientific) for 15 min. Coverslips were mounted on glass slides using Dako mounting media (Agilent Technologies, Santa Clara, CA, USA) and visualized using a Zeiss Axiovert 200 M fluorescence microscope (Carl Zeiss Light Microscopy, Göttingen, Germany). Images were captured using Zeiss imaging software and exported into Adobe Photoshop CS6 (Adobe, San Francisco, CA, USA) for cropping and minor adjustments.

### 2.10. RNAi Depletion

Transfections were performed as described above but with the following AllStars small interfering RNA (siRNA) duplexes (Qiagen): non-silencing (Scramble) siRNA (SI03650318), human ZEB1 siRNA (SI04272492), and human ZEB2 siRNA (SI02664277). All siRNAs were used at a final concentration of 20 nM.

### 2.11. Accession Number

Raw sequence reads for the cellular microRNA analysis from neurons and primary fibroblasts have been deposited in the National Center for Biotechnology Information (NCBI) BioSample database (accession number PRJNA357053).

## 3. Results

### 3.1. Increased Abundance of Host miR-182 and miR-183 during HSV-1 Infection

**Figure 1:**
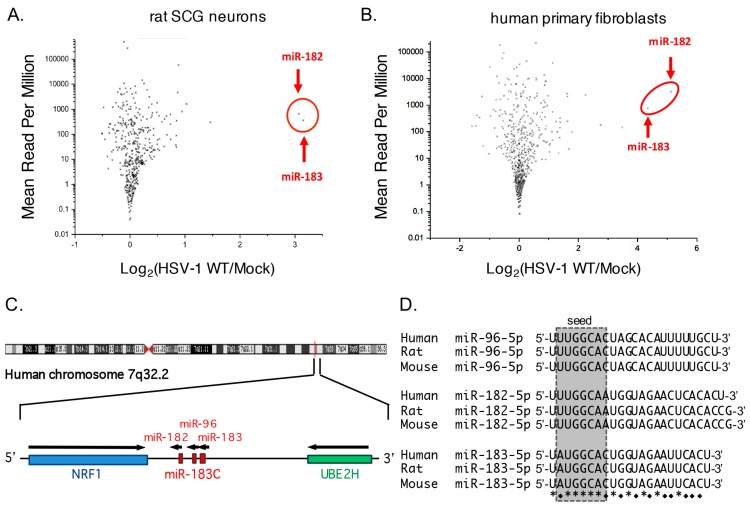
Induction of the host miR-183/96/182 cluster during herpes simplex virus 1 (HSV-1) infection of primary fibroblasts and neurons. (**A**) Scatter plot showing the ratio of normalized counts for individual host miRNAs from rat superior cervical ganglia (SCG)-derived neurons that were either mock infected or infected with wild type HSV-1 GFP-Us11 (strain Patton) at a multiplicity of infection (MOI) of 1 plaque forming unit (pfu) per neuron in the presence of acyclovir [42,43,44]. Under these conditions wild type HSV-1 establishes a quiescent infection within 5–7 days, wherein the viral genome is retained in up to half of the neurons but no infectious virus can be detected [43]. Seven days after infection, total RNA was collected and used to generate small-RNA libraries that were queried by deep sequencing. Data points corresponding to miR-182 and miR-183 are highlighted; (**B**) Comparison of individual host miRNA counts in a quiescent infection model using growth arrested human primary fibroblasts infected with HSV-1 wild type (WT) or mock-infected; (**C**) Schematic showing the long arm of human chromosome 7 and organization of the microRNA miR-183/96/182 gene (miR-183C) and flanking protein coding genes, nuclear respiratory factor 1 (NRF1) and ubiquitin-conjugating enzyme E2H (UBE2H). Arrow indicates transcriptional orientation; (**D**) Alignment of mature miR-96-5p, miR-182-5p, and miR-183-5p sequences from human, rat, and mouse. Shading indicates the predicted seed sequences. Invariant residues are indicated with an asterisk. Residues conserved in two of the three paralogs are indicated with a diamond. Both miR-96 and miR-183 are identical in each of three species, whereas human miR-182 differs slightly at the 3′-end from the rodent counterparts.

In an earlier study, we used deep sequencing to determine the relative expression of HSV-1 encoded miRNAs in three different cell culture models of latency or quiescent infection [46]. We found that infection of cultured rat SCG neurons infected with HSV-1 GFP-Us11 (strain Patton) produced a viral miRNA profile that was most similar to that of latently infected human ganglia. After subtracting low-quality sequences from the RNA-sequencing (RNA-seq) data, we mapped approximately 1.8 × 10^7^ reads onto the rat and HSV-1 genomes, of which 1.1 × 10^7^ (61%) could be assigned to cellular miRNAs (miRBase release 20) using sRNAbench [45,47]. Overall, the numbers and relative abundance of the miRNAs detected in the infected and uninfected samples were similar (Figure 1A), suggesting that HSV-1 infection does not cause global changes in host miRNA abundance. However, there were some exceptions, notably miR-182 and miR-183, which gave about 10-fold more reads in the infected cultures relative to mock-infected cultures.

To ask if this was specific to neurons, a similar analysis was performed using RNA-seq data from serum starved HFFs, i.e., non-neuronal primary cells, infected with wild type HSV-1 strain KOS at an MOI of ~1 and incubated at 42 °C for 72 h [46]. In this quiescent infection model, infectious particles are not detected but quiescent virus can be induced to reenter productive (lytic) replication by expression of ICP0 and the profile of viral miRNA accumulation is different to that of latently infected human ganglia [46,48]. A total of 9.2 × 10^6^ reads were mapped to the human and HSV-1 genomes, with 6.0 × 10^6^ (65%) corresponding to known human miRNAs. As with the latently infected rat SCG-derived neurons, the abundance of most cellular miRNAs was not changed significantly by viral infection (Figure 1B), but the number of reads mapping to miR-182 and miR-183 was increased substantially, here by 25- to 37-fold in the presence of HSV-1.

### 3.2. Robust Induction of the miR-183/96/182 Cluster during Acute HSV-1 Infection

The genomic versions of miR-182 and miR-183 are located close together on human chromosome 7 (Figure 1C), and form part of an evolutionarily-conserved miRNA cluster comprised of three paralogs transcribed from the same DNA strand in the following order: miR-183, miR-96, and miR-182 [49]. In primates including humans, the mature miR-183 and miR-96 sequences are separated by 136 bp, with miR-182 located 4.2 kb away. This arrangement is retained in rodents and other animals but with different spacing. Across all vertebrates, the sequences of the mature miRNAs are highly conserved (Figure 1D), both within and between the paralogs, implying a shared evolutionary origin and shared functions [49,50]. Imperfect conservation within the seed sequences results in the microRNAs having both common and unique target mRNAs.

All three miRNAs are co-expressed during embryogenesis, primarily in the developing sensory organs and associated neuronal ganglia, as well as in stem cells [51,52]. Expression of the cluster is generally low in differentiated tissues but is frequently deregulated in cancer [53], and in several autoimmune [54,55] and neurodegenerative disorders [56,57]. Recent studies have implicated the miR-183/96/182 cluster in development and differentiation of sensory neurons [51,52,58] and in epigenetic mechanisms controlling neuronal plasticity and pain responses [59,60]. In most contexts, all three miRNAs are co-transcribed as a single common primary (pri-) miRNA transcript that is subsequently processed into three separate precursor (pre-) miRNAs [52,61], but there is evidence from sarcoma metastasis models and tumor-derived cell lines for decoupled expression through internal promoter usage [62,63,64,65,66].

**Figure 2:**
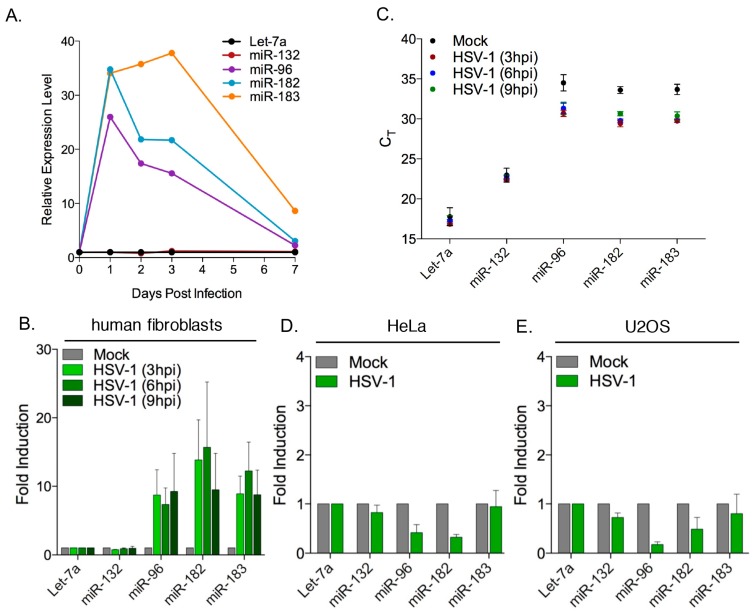
Levels of all three members of the miR-183 cluster are induced by HSV-1 infection of primary neurons and fibroblasts. (**A**) Time course analysis of mature Let-7a, miR-132, miR-96, miR-182, and miR-183 levels in rat SCG-derived neuron cultures pretreated with acyclovir (ACV) for one day (at day 6 in vitro) and then infected with HSV-1 wild type (GFP-Us11 strain Patton) at an MOI of 1 pfu/neuron in the presence of ACV. RNA was harvested at days 0, 1, 2, 3, and 7 post-infection and queried by SYBR green-based reverse transcription quantitative polymerase chain reaction (RT-qPCR), with values normalized to Let-7a; (**B**) RT-qPCR analysis of miR-132, miR-96, miR-182, and miR-183 levels in RNA isolated from normal human dermal fibroblasts (NHDFs) infected with HSV-1 wild type (MOI = 3) relative to mock infected controls. Samples were collected at 3, 6, or 9 h post-infection (hpi). Values are normalized to Let-7a and plotted as the mean and standard error of three biological replicates; (**C**) Raw C_t_ (cycle threshold) values for the data shown in panel B, verifying the selective induction of the miR-183 cluster miRNAs; (**D**,**E**) Analysis of host miRNA abundance in HeLa (**D**) and U2OS (**E**) cells after infection with HSV-1 wild type (MOI = 3) relative to mock-infected cells. Samples were collected at 6 h post infection.

In light of the placement of miR-96 between miR-183 and miR-182, the lack of reads corresponding to miR-96 in both deep sequencing analyses was puzzling and suggested an artifact of the analysis. To address this, we used a SYBR green based reverse transcription quantitative PCR (RT-qPCR) assay to quantify the levels of miR-96 with or without HSV-1 infection, and also provide validation of the observed increase in miR-183 and miR-182 levels using an independent method. Neuron cultures were infected with HSV-1 as above and RNA collected at 0, 2, 3, or 7 days post-infection (Figure 2A) corresponding to the period during which latency is established. Amplification products corresponding to all three members of the cluster, miR-183, miR-96, and miR-182, were readily detected and had increased substantially after one day of infection before dropping over the next six days. In contrast, levels of Let-7a, as well as miR-132, a neuronal miRNA reported to be upregulated in monocytes following HSV-1 infection [67], were essentially unchanged. Even at day 7 post-infection, the levels of miR-183, and to a lesser extent miR-182 and miR-96, remained elevated above the pre-infection baseline, further validating the increase in reads for miR-183 and miR-182 observed in the RNA-seq analysis and confirming that miR-96 is also induced.

To address lytic infection, NHDFs were acutely infected with HSV-1 and total RNA collected in triplicate at 3, 6, and 9 h post-infection (Figure 2B). The RT-qPCR analysis showed that levels of miR-183, miR-96, and miR-182 were each induced approximately 10-fold in the infected cells compared to mock infected, with the maximum increase achieved within 3 and 6 h. This increase is also evident from the *C*_t_ (cycle threshold) values (Figure 2C) and similar increases were observed in HFFs using an alternative stem-loop, Taqman-based RT-qPCR assay (I.J., A.L., and D.M.C, unpublished results). Interestingly, induction of the miR-183/96/182 cluster by HSV-1 was not observed in two highly transformed human cell lines, HeLa (Figure 2D) and U2OS (Figure 2E), and if anything levels of these miRNAs were reduced by infection relative to the uninfected controls. Both of these transformed cell lines were highly permissive for HSV-1 replication as indicated by GFP-Us11 expression, and are used extensively to characterize viral functions.

These results recapitulate the specific increases observed by RNA-seq and confirm that miR-96 is induced alongside miR-183 and miR-182. It is worth noting that others have reported under-representation of miR-96 relative to the other two paralogs [34]. Induction was most reliably observed following infection of non-transformed, primary cells (peripheral neurons and fibroblasts) and was readily detected by 3 h post infection.

### 3.3. Transcription of the miR-183/96/182 Cluster Is Stimulated by HSV-1

**Figure 3:**
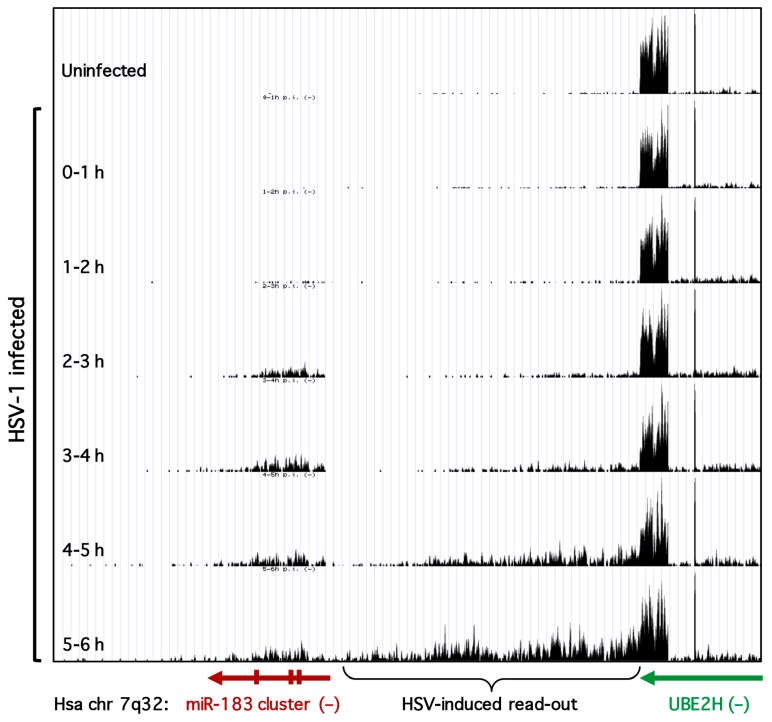
Transcription of the miR-183 cluster is induced during HSV-1 infection of primary fibroblasts. UCSC Genome Browser view of the human miR-183/96/182 cluster locus (negative strand, chromosome 7q32.2) and flanking region including the ubiquitin-conjugating enzyme E2H (UBE2H) gene showing the locations of 4sU-labeled RNA-seq reads obtained from primary human foreskin fibroblasts (HFFs) either uninfected or infected with wild type HSV-1 (strain 17) and harvested at various time points. Adapted from data (GEO database accession No. GSE59717) generated by Rutkowski, Dölken, and colleagues [27].

The analysis thus far measured levels of the mature microRNA products and does not distinguish between elevated transcription of the pri-miRNA, altered processing of the pri- and pre-miRNAs, or increased stability of the mature miRNAs. To address the first of these possibilities, we examined published genome-wide strand-specific RNA-seq data for nascent host transcripts produced during HSV-1 infection of HFFs [27]. A UCSC genome browser (https://genome.ucsc.edu/, University of California Santa Cruz, Santa Cruz, CA, USA) view of a region of human chromosome 7 centered on the miR-183/96/182 cluster is shown in Figure 3. For simplicity, only nascent RNA-seq reads mapping to the negative (−) strand are shown. In uninfected cells, very few reads are mapped to the miR-183/96/182 cluster consistent with the low levels of the mature miRNAs in these differentiated primary fibroblasts. However, within 2 to 3 h of infection, there is a clear increase in reads mapping across the cluster. This increases further over the next 2 h before dropping slightly. The profile is consistent with elevated transcription of the miR-183/96/182 locus at early times in HSV-1 infection, producing pri-miRNAs spanning all three miRNAs.

Profiling nascent host transcripts during HSV-1 infection, Dölken and colleagues showed there is extensive read-through beyond the poly(A) sites of many host genes, so-called “read-out” transcription [27]. An example of this unexpected phenomenon is clearly illustrated by the gene for the E2 ubiquitin-conjugating enzyme UBE2H that lies immediately upstream of the miR-183/96/182 cluster and is transcribed from the same DNA strand. The *UBE2H* gene is actively transcribed before and during HSV-1 infection but as the infection progresses there is a striking accumulation of reads mapping to the intergenic region immediately downstream of the UBE2H transcription unit. As noted by these authors, for genes that are not transcribed at high levels, the abnormal transcripts originating upstream of the actual transcription unit can be mistaken for induction. This does not appear to be the case for the miR-183/96/182 cluster because there remains an extended region with very few reads separating the miR-183/96/182 cluster from the UBE2H read-on transcripts at 2 and 5 h after infection when induction of the cluster occurs. Thus it appears that the selective increase in miR-183, miR-96, and miR-182 detected by deep sequencing and by RT-qPCR arises through transcriptional activation of the genomic locus, which is effectively silent in uninfected HFFs.

### 3.4. Induction of the miR-183/96/182 Cluster Requires Viral Gene Expression

**Figure 4:**
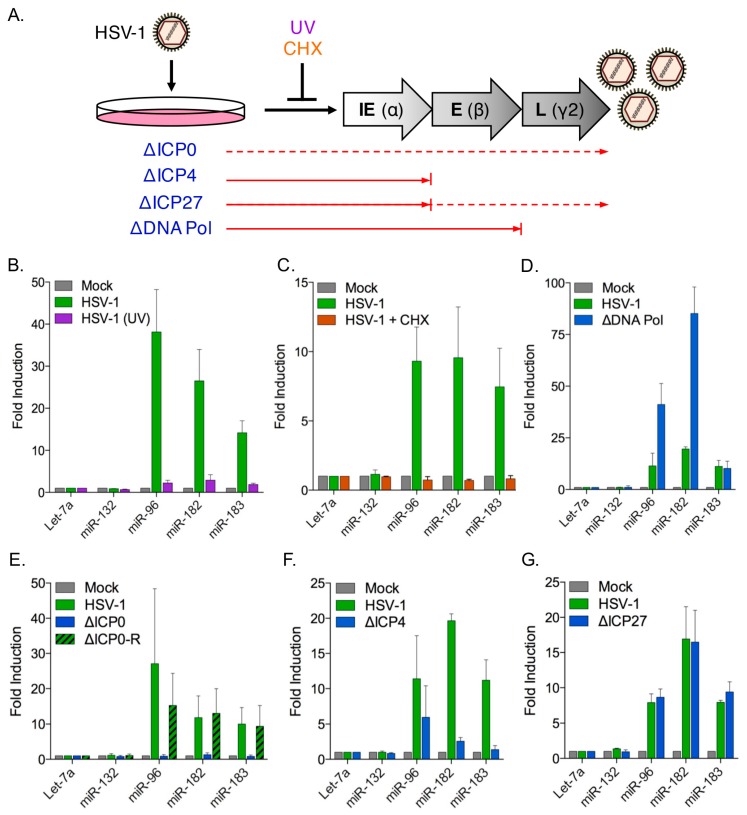
Induction of the host miR-183/96/182 cluster requires viral gene transcription and protein synthesis of an immediate-early viral factor. (**A**) Schematic representation of the HSV-1 productive (lytic) cycle gene expression cascade to illustrate the points of interruption caused by disruptive mutations in specific viral genes (ΔICP0, ΔICP4, ΔICP27, or ΔDNA polymerase) or exposure to ultraviolet (UV) radiation or protein synthesis inhibitor cycloheximide (CHX). Dashed arrows indicate reduced expression; (**B**–**G**) Profiling of cluster induction in NHDFs upon infection with (**B**) wild type or UV-irradiated HSV-1 wild type (GFP-Us11 strain Patton); (**C**) HSV-1 wild type in the presence or absence of CHX; (**D**) a ΔDNA polymerase (early) viral mutant relative to wild type HSV-1, and (**E**–**G**) immediate-early viral mutants (ΔICP0, ΔICP4, ΔICP27) relative to wild type HSV-1.

The HSV-1 productive cycle follows a sequential cascade involving three distinct waves of viral gene expression (Figure 4A) [68]. Transcription of the immediate early (IE) genes is stimulated by the viral tegument protein VP16 and the resulting IE proteins drive expression of the early (E) genes that are mainly involved in replication of the viral DNA genome. Replication of the genome stimulates transcription of the late (L) genes, which largely encode virion components and factors required for assembly and egress of virions.

To determine whether miR-183/96/182 induction requires HSV-1 gene expression, NHDFs were infected with either untreated or ultraviolet (UV)-irradiated wild type HSV-1 GFP-Us11 virus (Figure 4B). Fibroblasts infected with the irradiated virus showed no increase in miR-183/96/182 levels compared to the mock-infected cells, whereas non-irradiated virus showed considerable induction. Thus viral gene expression is essential to induce the miR-183/96/182 cluster. To ask if viral protein synthesis was necessary, infected cells were treated with cycloheximide (CHX), an inhibitor of the eukaryotic translation machinery (Figure 4C). No increase in miR-183/96/182 levels was observed in the presence of CHX indicating that new protein synthesis is required for induction of the locus. Together, these results indicate that both the transcription and translation of one or more viral genes is required to activate the miR-183/96/182 cluster.

### 3.5. The HSV-1 Immediate-Early Proteins ICP0 and ICP4 Are Necessary for Maximal miR-183/96/182 Cluster Induction

To identify the viral protein(s) responsible, we performed infection studies using HSV-1 mutant viruses lacking key IE and E genes required for progression through the productive replication cycle. First a virus, HP66, lacking much of the viral DNA polymerase gene (HSV-1 ΔDNA Pol) [39] was used to test the requirement for viral DNA replication and late gene expression (Figure 4D). Infection with this mutant induced even greater levels of miR-96 and miR-182 than the wild type virus, ruling out any importance of active viral genome amplification or high-level expression of true-late gene products.

The HSV-1 IE proteins contribute to regulation of viral gene expression. Of these, ICP0 acts as a general transactivator of viral genes from each kinetic class [36,69], ICP4 acts both as an activator of viral E and L genes and as a repressor of the IE genes [70,71] and ICP27 promotes the expression, nuclear export, and translation of certain unspliced E and L mRNAs [72]. Infections were performed with viral mutants that do not express ICP0, ICP4, or ICP27 (Figure 4E–G). Infection with the ΔICP0 virus 7134 (Figure 4E) did not result in miR-183/96/182 induction, whereas its rescued derivative ΔICP0-R (7134R), and the ΔICP27 virus (d27-1), induced the cluster to similar levels as wild type (Figure 4E,G). Infection with the nonsense mutant *n*12 (ΔICP4) virus reduced but did not entirely abolish induction (Figure 4F). Thus, it appears that expression of at least two viral proteins, ICP0, and to a lesser extent ICP4, is required for maximal induction of the miR-183/96/182 cluster.

### 3.6. ICP0 Is Sufficient to Induce the miR-183/96/182 Cluster

**Figure 5:**
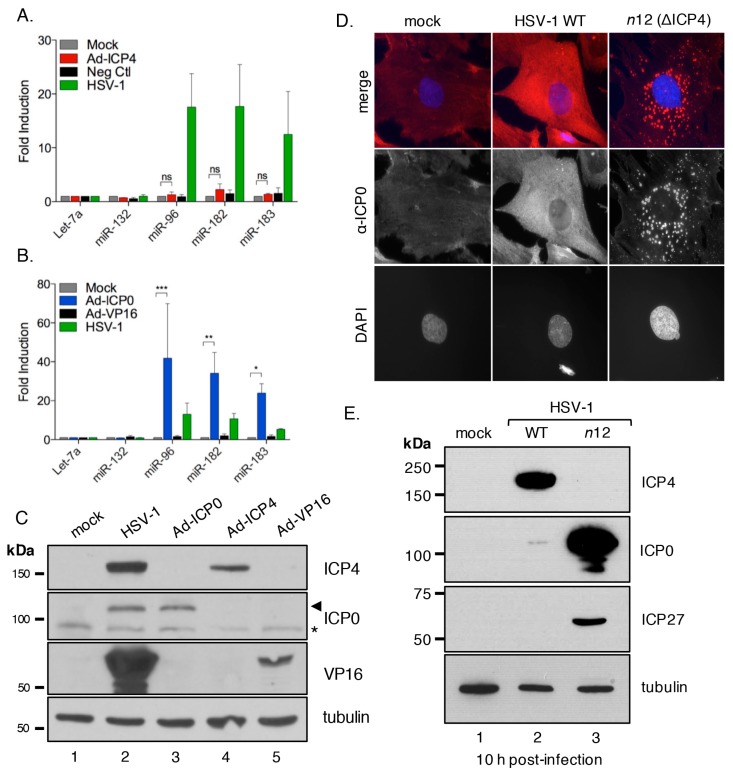
The immediate early viral factor ICP0 is sufficient to cause induction of the host miR-183/96/182 cluster. (**A**,**B**) Profiling of cluster induction relative to mock treated NHDFs, of adenoviral vectors delivering ICP0 (Ad-ICP0), ICP4 (Ad-ICP4), or VP16 (Ad-VP16) as a negative control. Lysates were prepared 48 h after infection. Statistical significance was calculated using a two-way analysis of variance (ANOVA) test, with * indicating a *p*-value of ≤ 0.05 and ** a *p*-value of ≤ 0.005, *** indicating a *p*-value of ≤ 0.0005, ns indicates not significant; (**C**) Immunoblot confirming expression of recombinant ICP0 (lane 3), ICP4 (lane 4), and VP16 (lane 5) from the adenoviral vectors. Infection with wild type HSV-1 serves a positive control (lane 2). ICP0 bands indicated by arrowhead, asterisk denotes a non-specific band. Mock (lane 1) and wild type HSV-1 (HSV-1 WT, lane 2) samples were collected 6 h post-infection, while all adenoviral samples were collected 48 h post infection; (**D**) Subcellular localization of ICP0 detected by indirect immunofluorescence in mock, wild type HSV-1, or ΔICP4 (*n*12) infections of NHDFs. Nuclei were visualized by 4′,6-diamidino-2-phenylindole (DAPI) staining; (**E**) Total protein was collected from mock, wild type HSV-1, and ΔICP4 (*n*12) infected NHDFs at 10 h post-infection and probed by immunoblotting with antibodies against viral immediate early proteins ICP4, ICP0, ICP27, and loading control, α-tubulin.

To ask if either ICP0 or ICP4 were capable of inducing the miR-183/96/182 cluster independent of HSV-1 infection each protein was expressed directly using a doxycycline-regulated adenoviral vector [40]. As a control, we expressed VP16, another viral transcriptional activator that is normally expressed as a leaky-late (γ1) gene, from the same adenoviral vector. As shown in Figure 5A, delivery of ICP4 did not result in any significant increase in miR-183/96/182 levels, whereas expression of ICP0 gave robust induction (Figure 5B). Expression of VP16 also resulted in no substantial increase. Expression of ICP0, ICP4, and VP16 from the adenoviral vectors was confirmed by immunoblotting (Figure 5C). Thus, we find that expression of ICP0 in the absence of any other HSV-1 gene products is sufficient to induce the miR-183/96/182 cluster.

### 3.7. ICP0 Must Be Nuclear to Induce miR-183/96/182

During the immediate-early phase of the HSV-1 productive replication cycle, ICP0 accumulates in the nucleus of infected cells but is later redistributed to the cytoplasm [73,74,75]. It has been shown previously that infection of Vero cells with viruses lacking functional ICP4 results in the aberrant cytoplasmic accumulation of ICP0 [76]. This is a consequence of the greatly increased levels of ICP27 caused by the loss of negative regulation by ICP4 [77,78]. Aberrant localization of ICP0 in fibroblasts might therefore explain why the ΔICP4 (*n*12) virus showed a reduced ability to induce miR-183/96/182 transcription, despite expression of wild type ICP0 (Figure 4F). To confirm that similar deregulation of ICP0 occurs in primary fibroblasts, we used indirect immunofluorescence to compare the localization of ICP0 during infection with wild type KOS and ΔICP4 (*n*12) viruses. As shown in Figure 5D, ICP0 synthesized by wild type virus was broadly distributed through the cytoplasm and nucleoplasm. Infection with ΔICP4 resulted in a strikingly different pattern of ICP0 localization, with the majority of the signal concentrated into discrete cytoplasmic foci, with little to no nuclear accumulation. Consistent with the lack of negative regulation by ICP4, the steady-state levels of both ICP0 and ICP27 were greatly elevated in the *n*12 mutant compared to wild type virus (Figure 5E). This shows that the previously described influence of ICP4 on ICP0 localization is recapitulated in primary cells and thus is not limited to transformed cells. It also explains the greatly reduced induction of the miR-183/96/182 cluster by the ΔICP4 (*n*12) virus.

**Figure 6:**
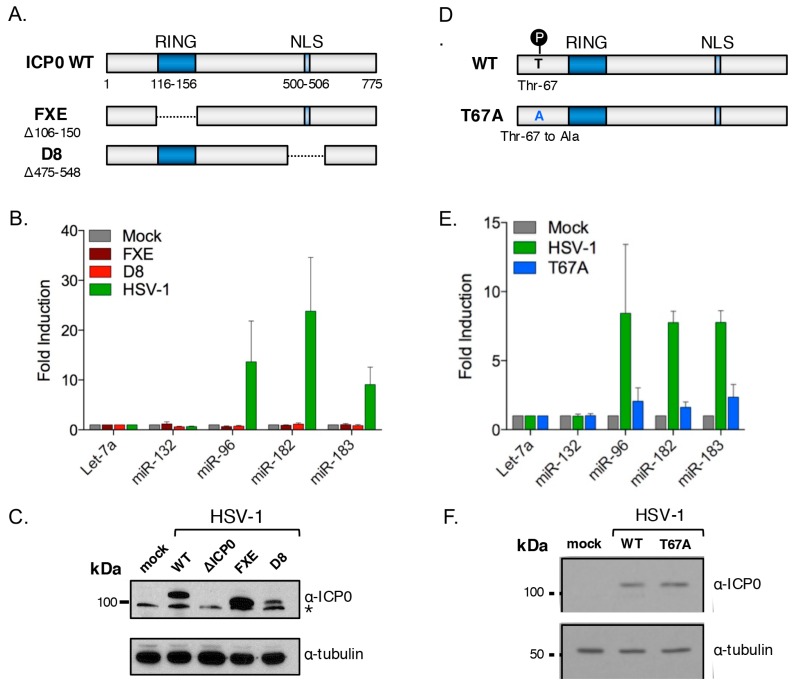
Induction of the host miR-183/96/182 cluster requires ICP0 nuclear localization and E3 ligase function to direct degradation of a host factor. (**A**) Schematic showing functional domains within wild type (WT) and mutant versions of HSV-1 ICP0. The RING (really interesting new gene) domain confers E3 ligase function to ICP0, and is deleted in the FXE mutant while the D8 mutant lacks an nuclear localization signal (NLS); (**B**) Profiling of cluster induction by WT HSV-1, FXE, and D8 virus infection of NHDFs; (**C**) Immunoblot detection of ICP0 expression by WT HSV-1 and respective mutant viruses. Asterisk indicates a non-specific band; (**D**) Schematic of ICP0 showing the location of phosphorylation residue threonine 67 and its substitution to alanine in the mutant form of ICP0 produced by the T67A virus; (**E**) Profiling of cluster induction by WT HSV-1 and T67A virus infection of NHDFs; (**F**) Immunoblot detection of ICP0 expression by WT and T67A HSV-1.

To further examine the role of ICP0 nuclear localization, infections were performed using the HSV-1 mutant virus D8 (Figure 6A), in which residues 475 to 548 encompassing the ICP0 nuclear localization signal (NLS, residues 500–506) are deleted [79]. Expression of the mutant ICP0 protein was readily detected, albeit at lower levels than from the wild type virus, and as predicted, the D8 virus was unable to induce the miRNA cluster (Figure 6B). This finding, combined with the behavior of the ΔICP4 virus, indicates that nuclear localization of ICP0 is required for it to stimulate transcription of the miR-183/96/182 transcription unit.

### 3.8. Cluster Induction Requires the E3 Ligase Function of ICP0

Many ICP0 functions involve the targeting of host proteins for accelerated degradation by the proteasome [69]. In many but not all cases, this requires the C3HC4 RING (really interesting new gene) domain (residues 116–156), which is required for E3 ubiquitin ligase activity [80,81]. To determine whether the ICP0 E3 ligase function was required for cluster induction, REFs were infected with HSV-1 FXE, a virus expressing a mutant version of ICP0 that lacks the RING domain (Figure 6A) [79]. As shown in Figure 6B, infection with this mutant failed to increase miR-183/96/182 levels, indicating a strict requirement for the RING domain and therefore the E3 ubiquitin ligase function.

Aside from the C3HC4 RING domain and NLS, other regions of ICP0 are involved in substrate recognition, often through direct protein–protein interactions [82,83,84,85]. In some cases, these interactions are dependent on post-translational modifications such as phosphorylation [86]. To further map the domain requirements for ICP0-mediated induction of the miR-183/96/182 cluster we tested a mutant virus HSV-1 T67A, in which threonine-67 of ICP0 is changed to alanine (Figure 6D) [86]. When this threonine residue is phosphorylated, ICP0 can interact with RNF8, a cellular E3 ubiquitin ligase involved in the double-stranded DNA break response. Infection with the T67A virus resulted in a very weak induction of the miR-183/96/182 cluster (Figure 6E) and the equal expression of the wild type and mutant proteins was confirmed by immunoblotting (Figure 6F). This suggests that phosphorylation of ICP0 at this position is required for full induction.

### 3.9. Transcriptional Repressor ZEB1 Is Targeted for Degradation by ICP0

**Figure 7:**
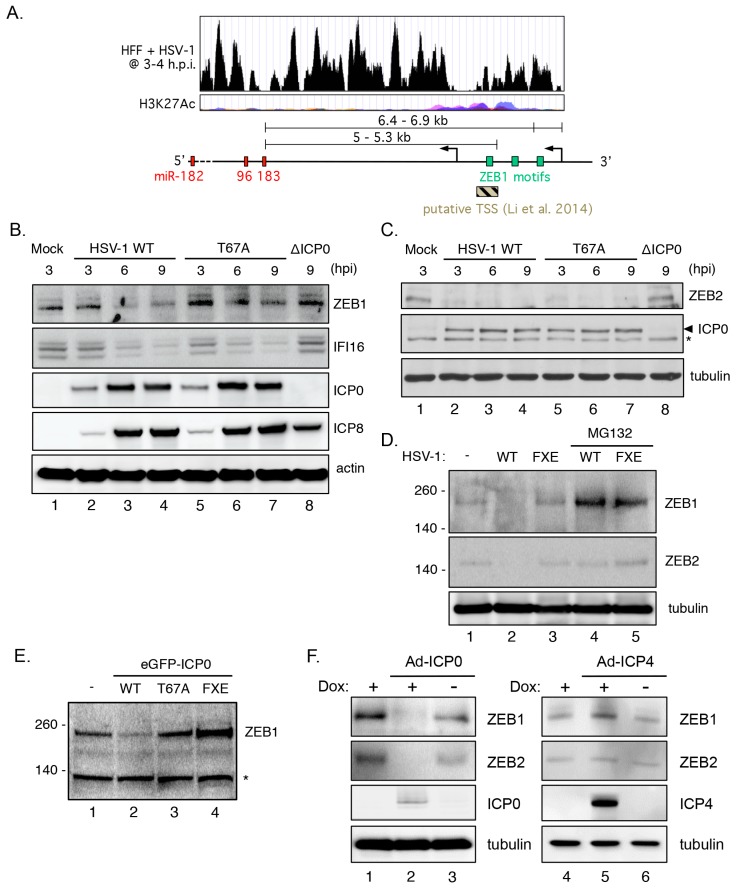
Induction of the host miR-183/96/182 cluster correlates with the degradation of host zinc finger E-box homeodomain (ZEB) transcriptional repressors by ICP0. (**A**) UCSC Genome Browser data from Rutkowski and colleagues [27] showing RNA-seq reads from human foreskin fibroblasts (HFFs) infected with wild type HSV-1 (HSV-1 WT) that map to the human miR-183/96/182 cluster locus and region upstream of the cluster. ENCODE data for the corresponding regions shows a region enriched for acetylated histone H3 (H3K27Ac). These regions are near sites posited to possess ZEB1 binding motifs as well as near a putative miR-183/96/182 cluster, transcription start site (TSS), as reported [53]; (**B**) Time course analysis of NHDFs infected with HSV-1 wild type (lanes 2–4) or T67A (lanes 5–7). Lysates were prepared at 3, 6, and 9 h post-infection. As controls, cells were either mock infected (lane 1) or infected with HSV-1 ΔICP0 (lane 8). Blotting was performed with antibodies against ZEB1, IFI16, ICP0, ICP8, and actin. Asterisk indicates a non-specific band; (**C**) Similar experimental setup as in B; blotting was performed using antibodies against ZEB2, ICP0, and tubulin. A non-specific band detected by the ICP0 antibody is marked with an asterisk; (**D**) Analysis of ZEB1 and ZEB2 levels in HFF cells either mock infected or infected with HSV-1 wild type (lanes 2 and 4) or FXE (lanes 3 and 5). For lanes 4 and 5, the proteasome inhibitor MG132 was added to the culture media prior to harvest at 8 h post infection; (**E**) Immunoblotting of ZEB1 in 293T cells transfected with plasmids expressing the wild type, T67A, and FXE forms of ICP0. Asterisk indicates a non-specific band; (**F**) HFF cells were infected with adenoviral vectors encoding ICP0 (Ad-ICP0, lanes 2 and 3) or ICP4 (Ad-ICP4, lanes 5 and 6) together with Ad.C-rtTA, an adenovirus expressing the doxycycline regulated transactivator. After infection, doxycycline was either added (+) or omitted (−) from the media.

Transcriptional regulation of the miR-183/96/182 cluster is complex and dependent on cell context [49]. Two potential transcriptional start sites (TSSs, Figure 7A) for the precursor transcript induced during HSV-1 infection were evident in the RNA-seq analysis [27]. These were located between 5.0 and 6.9 kb upstream of the mature miR-183 sequence and within or just upstream of a region enriched for acetylated histone H3 lysine-27 (H3K27Ac), a feature of enhancer regions [87]. This region includes previously reported TSSs identified in tumor cell lines displaying dysregulated expression of the miR-183/96/182 cluster [33,53,88,89]. Three matches to the consensus binding sequences for the ZEB family of transcription factors are found in this region. ZEB1 (also known as δ-crystallin enhancer binding factor 1 (δEF1) or transcription factor 8 (TCF8) is implicated in the repression of a wide-array of genes including the miR-183/96/182 cluster [53]. Moreover, both ZEB1 and ZEB2 (also named Smad-interacting protein 1 (SIP1)), a related transcriptional factor with very similar DNA binding specificity, were previously identified as SUMOylated proteins that are destabilized by ICP0 in HepaRG hepatocyte progenitors [90].

The impact on HSV-1 infection on ZEB1 abundance in HFFs was assessed by immunoblotting (Figure 7B). With an apparent molecular weight of 190–210 kDa and low abundance in primary fibroblasts, ZEB1 is relatively difficult to blot, however two prominent bands were detected using a ZEB1 specific antibody. Levels of both bands were reduced in the presence of wild type HSV-1 (Figure 7B, lanes 2–4) compared to mock infected cells (lane 1) or cells infected with HSV-1 ΔICP0 (lane 8). Infection with HSV-1 T67A had a more modest effect on ZEB1 levels (lanes 5–7), consistent with its incomplete effect on miRNA induction. In contrast, the levels of IFI16, a known degradation substrate of ICP0, were reduced by infection both the wild type and T67A viruses. Detection of the viral ICP0 and ICP8 proteins provided controls for each of the infections. Levels of ZEB2 were also strongly reduced by infection with the wild type (lanes 2–4) or T67A viruses (lanes 5–7) but not by HSV-1 ΔICP0 (lane 9) (Figure 7C). Similarly, treatment of HSV-1 infected cells with proteasome inhibitor MG132 added after the onset of the infection was sufficient to stabilize ZEB1, but interestingly not ZEB2, in the presence of HSV-1 expressing either wild type ICP0 or the FXE mutant (Figure 7D).

The reduction in ZEB1 levels could be recapitulated by transfection of a plasmid expressing wild type ICP0 fused to enhanced GFP (eGFP-ICP0, Figure 7E), showing that ICP0 is sufficient for turnover of ZEB1. Reductions in ZEB1 were blocked either by the T67A mutation or by removal of the RING domain required for E3 ligase function (FXE). Likewise, expression of ICP0 using the dox-regulated adenoviral vectors shown earlier to induce miR-183/96/182 expression (Figure 5B) resulted in the loss of detectable ZEB1 and ZEB2 (compare lanes 2 and 3, Figure 7F). In contrast, levels were not altered by adenoviral expression of ICP4 (lanes 4–6, Figure 7F). Together these results show that ZEB1 and ZEB2 are destabilized by ICP0. For ZEB1, at least, this requires both the resident E3 ubiquitin ligase function of ICP0 and host proteasome activity.

### 3.10. ZEB1 Suppresses miR-183/96/182 Cluster Transcription in Primary Fibroblasts

**Figure 8:**
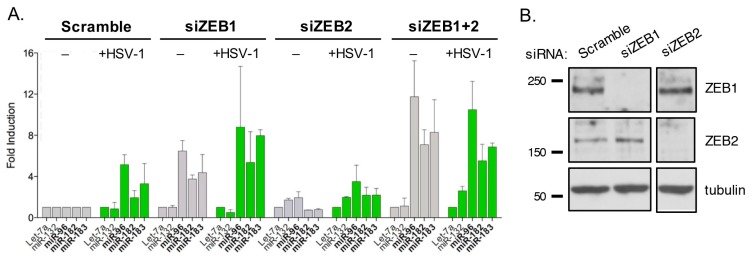
ZEB factors regulate miR-183/96/182 cluster expression. (**A**) Profiling of Let-7a, miR-132, miR-96, miR-182, and miR-183 levels in NHDFs either mock (gray bars) or wild type HSV-1 (green bars) infected after transfection with non-specific (Scramble) and specific (ZEB1, ZEB2) siRNAs. The means and standard error are shown. (**B**) Immunoblot to confirm knockdown of ZEB1 and ZEB2 in NHDFs transfected with anti-ZEB small interfering RNAs (siRNAs).

Given that ZEB1 and ZEB2 can act as gene-specific repressors and that their levels are reduced in HSV-1 infected cells, we asked if depletion of ZEB1 and/or ZEB2 using siRNAs was sufficient to elevate levels of miR-183, miR-96, and miR-182 in the absence of HSV-1 or ICP0 (Figure 8A). NHDFs were transfected individually or in combination with siRNAs against ZEB1 and/or ZEB2 or with a control siRNA (Scramble). The effectiveness of the specific siRNAs was confirmed by immunoblotting (Figure 8B). Depletion of ZEB1 resulted in a clear increase in the miR-183/96/182 cluster miRNAs, whereas depletion of ZEB2 had little to no effect (Figure 8A). Interestingly, simultaneous depletion of both factors (siZEB1+2) resulted in a somewhat greater increase than for knockdown of ZEB1 alone, suggesting some level of redundancy between the two ZEBs.

The effect of the siRNAs was enhanced to a modest level by coinfection with wild type HSV-1 (Figure 8A). It should be noted that the transfection conditions greatly reduce the infectivity of HSV-1, presumably by establishing an antiviral state, and as a consequence, the fold increase in miR-183/96/182 levels was reduced compared to other infection experiments shown in this study. Regardless, the results indicate that transcriptional repression ZEB1 and ZEB2 contribute to the very low expression of miR-183, miR-96, and miR-182 in primary fibroblasts.

## 4. Discussion

Viral infection can have a profound effect on cellular gene expression with host shutoff being the most extreme manifestation. This can benefit the host by creating a less permissive environment for viral replication or alternatively, benefit the virus by disarming innate defenses such as the interferon pathway [91,92] and by sharpening transitions between different classes of temporally transcribed mRNAs [24,25]. By reducing the stability and translation of multiple mRNAs, miRNAs are widely used by animal cells to coordinate the output of gene networks and would seem to be logical targets for manipulation by viruses [32]. In this study, we have shown that there are in fact few large-scale changes in the abundance of host miRNAs during HSV-1 infection of primary fibroblasts and peripheral neurons. The notable exception is the 10-fold or greater increase in three miRNA paralogs: miR-96, miR-182, and miR-183, brought about by transcriptional activation of a shared chromosomal locus. Using mutant viruses and the expression of individual HSV-1 factors we have clearly demonstrated that the HSV-1 IE protein ICP0 is both necessary and sufficient to induce the miR-183/96/182 cluster; a new property for this intensively studied viral regulatory factor. Induction of these three miRNAs is reminiscent of, but at the same time distinct from, the 10-fold or greater induction of the non-coding telomere repeat containing RNA (TERRA) by HSV-1 [93]. Combined with the disruption of other telomere components, TERRA expression promotes the repurposing of host telomeres to form ICP8-associated pre-replication foci necessary for viral genome amplification. Although the E3 ligase function of ICP0 is necessary for activation of TERRA transcription, additional viral activities are required for full effect.

Following completion of the studies described here, the Booth Laboratory reported the altered regulation of 78 unique host miRNAs in the brains of a mouse model for acute HSV-1 encephalitis, including elevated expression of both miR-182 and miR-183 [94]. These changes (24 miRNAs increased by more than 2.5-fold and 54 miRNAs decreased) coincided with a general reduction in read numbers mapping to known host miRNA. There is a strong inflammatory response in this model and the samples contain mononuclear infiltrates and other cell types. However, in situ hybridization demonstrated an upregulation of miR-182 in neurons, although interestingly this was not limited to neurons infected with HSV-1. The direct involvement of viral factors in deregulation of host miRNA expression was not addressed. Nonetheless, these observations provide in vivo support for our observations in cultured cells.

**Figure 9:**
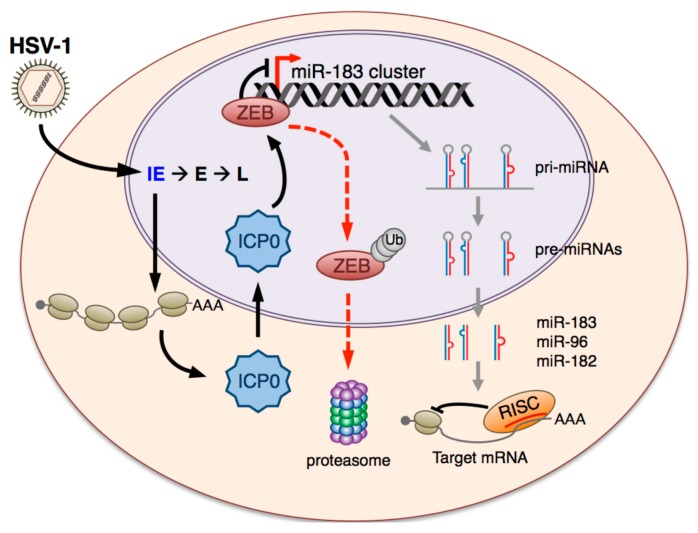
Proposed mechanism for upregulation of the miR-183/96/182 cluster by HSV-1 in non-transformed, terminally differentiated cells such as primary fibroblasts and neurons. Prior to infection, the miR-183/96/182 locus is transcriptionally repressed by the ZEB1 and ZEB2 transcription factors. Upon infection by HSV-1, the immediate early protein ICP0 is synthesized and imported into the nucleus, where it acts as an E3 ubiquitin-ligase to promote degradation of the ZEB proteins by the proteasome. This leads to de-repression of the transcriptionally silenced miR-183/96/182 locus and increased synthesis of the pri-miRNA transcript, which is subsequently exported and processed to produce the mature miR-183, miR-96, and miR-182 miRNA, which may become functional after incorporation into the RNA-induced silencing complex (RISC).

Our model for the induction of the miR-183/96/182 locus during HSV-1 infection is illustrated in Figure 9. In differentiated cells such as fibroblasts or neurons, the miR-183/96/182 locus is transcribed at very low levels due to the action of chromatin-associated transcriptional repressors such as ZEB1 and ZEB2 acting through corepressors which include C-terminal binding protein 1 (CtBP1), C-terminal binding protein 2 (CtBP2), and histone deacetylase 2 (HDAC2) [34]. After synthesis, ICP0 is imported into the nucleus where the E3 ubiquitin ligase activity associated with the ICP0 RING finger domain targets the resident ZEB proteins promoting degradation by the proteasome. This may involve direct ubiquitylation of the ZEB proteins by ICP0 but further study is needed. The reduced levels of ZEB1, and perhaps ZEB2, stimulate transcription of the miR-183/96/182 locus producing pri-miRNA transcripts that are processed and exported into the cytoplasm where they undergo further maturation and loading into the RNA-induced silencing complex (RISC).

Although ICP0 is dispensable for replication in cell lines lacking a functional antiviral response, it is required for acute infection in most other cells and for reactivation in explanted ganglia from latently infected mice [95,96,97,98]. A number of different functions have been assigned to ICP0, including the degradation and dispersal of proteins involved in intrinsic antiviral resistance [69,99,100] and in chromatin-mediated silencing [101,102]. Many targets for ICP0-mediated degradation are SUMO-conjugated proteins [103,104], and stable isotope labeling with amino acids in cell culture (SILAC)/mass spectrometry analysis of 877 cellular SUMOylated proteins in HepaRG hepatocytes found that 30% showed a change in abundance during HSV-1 infection [90]. ZEB1 and ZEB2 are known SUMO2 substrates and their abundance was reduced 5–7 fold and 3–5 fold respectively upon HSV-1 infection of HepaRG cells [90]. Our data supports this observation and provides direct evidence that ZEB1 and ZEB2 are ICP0 targets. To our knowledge, this is the first example of gene specific transcriptional regulation by ICP0 through the degradation of specific host transcriptional repressors.

The fact that we observed induction of the miR-183/96/182 cluster in models of latency seems contradictory given that ICP0 is classified as an IE gene product and expressed during the productive (lytic) cycle. However, this temporal assignment may not be so clear-cut. There is genetic evidence that ICP0 is expressed in murine sensory neurons during the establishment of latency, such that mutations that either increase or decrease ICP0 expression lead to changes in the structure of viral chromatin and in the expression of both productive-cycle genes and the latency-associated transcript (LAT) RNA [12,101,102]. In agreement, ICP0 transcripts can be detected in latently infected ganglia from mice [12,105,106] and there is evidence from neuronal marking studies that many latently infected neurons have at some point activated the ICP0 promoter [107,108]. It is worth emphasizing that abundance of the miR-183 cluster in the SCG neuron infection model at day 7 was much lower than over the first three days of infection. Over the time scale of these experiments, ZEB levels may be reduced by ICP0 expression at the beginning of the infection and not return to baseline before the cells are harvested. The miR-183 cluster miRNAs participate in a negative feedback loop with ZEB1 and ZEB2, each suppressing the expression of the other because of binding sites for miR-183/96/182 cluster members in the 3′ untranslated regions (UTRs) of the ZEB1 and ZEB2 mRNAs [33,109,110]. This self-reinforcing loop would maintain elevated miR-183/96/182 expression by preventing the synthesis of new ZEB proteins.

At this time, we can only speculate on the biological impact of microRNA upregulation with respect to HSV-1. The miR-183 family targets a diverse set of host mRNAs, some of which have the potential influence viral replication by changing the behavior or resilience to stress of the infected cells [49]. We have not identified any viral mRNAs that might serve as obvious targets. It is also possible that lowering the abundance of the ZEB proteins is a more important objective and that changes to the host microRNA repertoire is merely a consequence of removing these pre-existing repressors. These and other questions will be addressed in due course.

Interestingly, HSV-1 is not the only herpesvirus to alter expression of the miR-183/96/182 cluster or interact with the ZEB proteins. HCMV, a β-herpesvirus, alters the abundance of several host miRNAs during its productive infection cycle, with miR-96, miR-182, and miR-183 being the most strongly upregulated [111,112]. β-Herpesvirueses lack a true ICP0 homolog, however the HCMV immediate early protein IE1 and tegument protein pp71 can functionally compensate for ICP0 in supporting the replication of an HSV-1 ICP0-null virus [113]. Whether the ZEB proteins are targeted in some way during HCMV infection is being tested.

During type II and III latency, the γ-herpesvirus EBV selectively downregulates miR-183/96/182 expression [114,115,116]. This is mediated by the viral latent membrane protein I (LMP-1), the major oncoprotein of EBV [114]. Reduced miR-183/96/182 expression helps push EBV-infected B-lymphocytes and epithelial cells towards a more mesenchymal phenotype, although additional viral and host miRNAs contribute to this phenotypic shift [117,118]. ZEB1 and ZEB2 have been clearly shown to antagonize EBV lytic gene expression by directly binding to the immediate-early BZLF1 promoter and possibly also the BRLF1 promoter, preventing expression of these essential transcriptional initiators [119,120]. Mutation of the ZEB binding sites in the BZLF1 promoter results in a virus that is predisposed to spontaneous reactivation [120], and overexpression of ZEB1 represses BZLF1 promoter activity from a plasmid or its natural context in the EBV genome [121].

Collectively, these studies reveal a recurring interaction between representatives of the three major herpesvirus subfamilies and a conserved host regulatory network involving the ZEB factors and ZEB-regulated genes such as the miR-183 cluster. More study is needed to understand the functional consequences for each virus and this may depend on the infected tissue, input from other signaling pathways, and whether the virus is replicating productively or maintaining latency. For HSV-1, we have defined a mechanism for manipulation the ZEB factors via the viral E3 ligase and shown that even in the context of a robust host shut off, HSV-1 can selectively modify the profile of host miRNAs.
